# Disturbance of Growth in Pediatric Patients Due to Osteomyelitis Caused by Growth Plate Infection

**DOI:** 10.7759/cureus.50631

**Published:** 2023-12-16

**Authors:** Jamal Malik, Syed Khaled

**Affiliations:** 1 Department of Research, Liberty University College of Osteopathic Medicine, Lynchburg, USA; 2 Gastroenterology, North Kansas City Hospital, Kansas City, USA

**Keywords:** growth cessation, bone growth, osteo-myelitis, osteo, infection, s. aureus, hematogenous, growth plate, pediatrics, osteomyelitis

## Abstract

Osteomyelitis, a severe bone infection, poses a multifaceted challenge to healthcare professionals. While its pathophysiology and treatment have been extensively studied, the impact of osteomyelitis on skeletal growth, particularly in pediatric patients, is an area that warrants attention. This abstract highlights the significance of understanding and managing growth disturbances in osteomyelitis, providing key findings and recommendations for clinicians. Understanding growth disturbance in osteomyelitis is essential because it can lead to lifelong consequences for pediatric patients. The infection may affect the growth plate, leading to limb length discrepancies, angular deformities, and functional impairments. These complications not only diminish the quality of life but also pose a substantial economic burden on the healthcare system. Therefore, early recognition and intervention are crucial. Key findings indicate that the risk of growth disturbances in osteomyelitis is particularly high in pediatric patients due to the vulnerability of the growth plate. Timely diagnosis, appropriate management, and targeted interventions can mitigate the long-term sequelae of growth disturbances. These include utilizing advanced imaging techniques to assess the extent of growth plate involvement, optimizing antibiotic therapy, and employing surgical techniques like epiphysiodesis, guided growth, or corrective osteotomies. Additionally, fostering a multidisciplinary approach that involves orthopedic surgeons, infectious disease specialists, and pediatric endocrinologists is vital to achieving successful outcomes. Recommendations for managing growth disturbance in osteomyelitis encompass early detection, meticulous monitoring, and a tailored treatment plan. Healthcare providers should remain vigilant for signs of growth plate involvement in osteomyelitis patients, especially in the pediatric population. A thorough evaluation, including advanced imaging and clinical assessment, is essential for accurate diagnosis. Close collaboration between specialists to address the infection and its skeletal consequences is crucial. Furthermore, patient and family education plays a pivotal role in fostering compliance with the treatment regimen. In conclusion, understanding and managing growth disturbances in osteomyelitis is paramount, particularly in pediatric patients. The implications of growth plate involvement are significant, and timely intervention is essential to prevent lifelong consequences. By implementing a comprehensive approach that combines accurate diagnosis, multidisciplinary collaboration, and patient education, healthcare professionals can enhance the quality of life and well-being of those affected by this challenging condition.

## Introduction and background

Growth disturbances in osteomyelitis

Osteomyelitis is a serious and potentially debilitating bone infection characterized by the inflammation of the bone and its marrow components. While its devastating effects on bone health and overall well-being have been widely recognized, the impact of osteomyelitis on skeletal growth, particularly in pediatric patients, is an aspect that has received increasing attention in recent years [1,2]. This introduction and background section aims to provide an overview of the significance of understanding and managing growth disturbances in osteomyelitis. Osteomyelitis poses a significant challenge to healthcare professionals, characterized by the complex interplay of host defenses, bacterial pathogens, and the bone itself. The infection typically originates from hematogenous spread, direct inoculation, or contiguous spread from adjacent tissues, leading to bone necrosis and the formation of sequestrae. Its pathophysiology, diagnosis, and treatment modalities have been the focus of extensive research, resulting in improvements in the management of the infection [3]. However, it is the secondary consequences of osteomyelitis, specifically its impact on skeletal growth, that have only recently gained the recognition they deserve. Pediatric patients, in particular, are highly susceptible to these growth disturbances due to the vulnerability of their growth plates [4]. The growth plate, also known as the physis, is a cartilaginous structure located at the ends of long bones. It plays a pivotal role in longitudinal bone growth by allowing the bone to elongate during the growth phase. This critical structure is particularly susceptible to the pathological processes associated with osteomyelitis, making pediatric patients especially prone to skeletal growth impairments [5,6]. The consequences of growth disturbances in osteomyelitis are profound. In cases where the growth plate is affected, patients may experience limb length discrepancies [7], angular deformities, and functional impairments, all of which can have a lasting impact on their quality of life [8]. Additionally, the economic burden associated with the long-term care and rehabilitation of individuals affected by osteomyelitis-related growth disturbances is substantial. Recognizing the importance of understanding and managing growth disturbances in osteomyelitis is crucial for improving patient outcomes. While the diagnosis and treatment of the underlying infection remain paramount, addressing the secondary complications related to growth plate involvement is equally essential. Early recognition, accurate diagnosis, and appropriate interventions can mitigate the long-term sequelae and ensure that patients can lead healthier, more productive lives. Therefore, this review article aims to shed light on the existing knowledge, key findings, and recommendations regarding growth disturbances in osteomyelitis, emphasizing the need for a comprehensive approach that encompasses both infection control and skeletal health.

## Review

Pathophysiology

Osteomyelitis is a bone infection with a complex pathophysiology that can lead to growth disturbances, particularly in the pediatric population [9]. Understanding the pathophysiological mechanisms underlying these growth impairments is essential for effective diagnosis and management. This section explores the pathophysiological aspects of growth disturbances in osteomyelitis. Osteomyelitis often begins with the introduction of pathogenic microorganisms into the bone. This can occur through various routes, including hematogenous spread, direct inoculation due to trauma or surgery, or contiguous spread from adjacent soft tissues. In pediatric patients, hematogenous spread is more common, with bacteria reaching the bone through the bloodstream. The bacteria most frequently responsible for osteomyelitis include *Staphylococcus aureus*, *Streptococcus* species, and *Haemophilus influenzae* [9]. Once the pathogenic microorganisms establish themselves within the bone, an intense inflammatory response is triggered. This response involves the activation of immune cells, such as neutrophils and macrophages, and the release of proinflammatory cytokines and chemokines. The inflammatory process can cause local tissue damage, leading to bone necrosis and the formation of sequestrae [10]. The infection and associated inflammation compromise the integrity of the bone. The release of enzymes and toxins by invading bacteria can result in bone destruction. In the context of growth disturbances, the most critical aspect is the involvement of the growth plate, also known as the epiphyseal plate or physis [11].

The growth plate is a specialized cartilaginous structure located at the ends of long bones, where longitudinal growth occurs. The bacterial infection can affect the growth plate directly or indirectly through the release of toxins, inflammatory mediators, and vascular compromise [11]. The involvement of the growth plate is a key factor in the pathophysiology of growth disturbances in osteomyelitis. The growth plate's unique characteristics, such as its rich blood supply and active cell turnover, make it particularly susceptible to bacterial invasion [12]. Infection of the growth plate can lead to its destruction and premature closure, disrupting the normal process of bone growth. This can result in limb length discrepancies, angular deformities, and functional impairments in pediatric patients [13]. In osteomyelitis, infection and inflammation can lead to vascular compromise within the affected bone. This compromise affects the blood supply to the growth plate, further exacerbating the damage. Insufficient blood flow can lead to ischemic changes, contributing to growth plate destruction and the formation of avascular areas within the bone [14]. The immune system's response to osteomyelitis includes the recruitment of immune cells and the formation of pus within the affected bone. This pus, along with dead tissue and debris, can create an environment conducive to bacterial growth and perpetuate the infection. In pediatric patients, the immune response can be heightened, as their immune systems are actively engaged in growth and development [14]. Understanding the pathophysiology of growth disturbances in osteomyelitis highlights the need for early diagnosis and appropriate management to prevent long-term sequelae. Accurate diagnosis and treatment should not only focus on eradicating the infection but also on addressing the secondary consequences, particularly the growth plate involvement. A comprehensive approach that combines infection control and measures to promote skeletal health is essential to ensuring optimal outcomes for patients affected by osteomyelitis-related growth disturbances [15].

Diagnosis

Diagnosing growth disturbances in osteomyelitis can be a complex process, as it involves recognizing both the underlying bone infection and its impact on skeletal development [16]. Timely and accurate diagnosis is crucial for implementing appropriate management strategies to mitigate long-term sequelae. This section discusses the diagnostic modalities and considerations for identifying growth disturbances in osteomyelitis. Clinical evaluation is the first step in the diagnostic process. Healthcare providers should maintain a high index of suspicion for osteomyelitis in pediatric patients, particularly when there is a history of predisposing factors such as recent trauma, surgery, or systemic illnesses [16]. Common clinical findings may include localized pain, swelling, erythema, warmth over the affected bone, and limited range of motion. Furthermore, pediatric patients may exhibit signs of growth disturbances, such as limb length discrepancies or angular deformities [16]. Imaging plays a pivotal role in diagnosing osteomyelitis and assessing its impact on skeletal growth. Several imaging modalities are useful, such as X-rays, MRIs, CT, ESR/CRP, and biopsy/culture. Conventional X-rays are often the initial imaging study, as they can reveal bony changes such as periosteal reactions, lytic or sclerotic lesions, and soft tissue swelling. In pediatric patients, growth plate involvement, epiphyseal irregularities, or asymmetry in bone length may be evident [15]. MRI is highly sensitive in detecting osteomyelitis and its complications. It provides detailed images of bone, soft tissue, and the growth plate. The presence of abscesses, sequestrae, and growth plate abnormalities can be identified through MRI [16]. CT scans are valuable for assessing bone involvement, sequestrae, and cortical bone defects. They are particularly useful for surgical planning in cases where bone deformities and growth plate disturbances require intervention [16]. Laboratory tests can aid in the diagnosis of osteomyelitis, although they are not specific to growth disturbances. Elevated inflammatory markers, such as erythrocyte sedimentation rate (ESR) and C-reactive protein (CRP), are often observed in cases of osteomyelitis. Blood cultures may also be performed to identify the causative pathogen [17]. When imaging and clinical findings strongly suggest osteomyelitis, a bone biopsy may be necessary to confirm the diagnosis and guide antibiotic therapy. Biopsy specimens are sent for culture and sensitivity testing to identify the infecting microorganism and determine its susceptibility to antibiotics [18]. In cases where growth disturbances are suspected, a specific assessment of the growth plate may be necessary. This can be achieved through advanced imaging techniques, such as MRI, which can provide detailed information on growth plate integrity and any pathological changes [16]. Pediatric patients with osteomyelitis and growth disturbances require a multidisciplinary approach to diagnosis and management. Collaboration between orthopedic surgeons, infectious disease specialists, and pediatric endocrinologists is crucial for comprehensively evaluating the patient's condition and formulating an appropriate treatment plan. Early diagnosis and recognition of growth plate involvement can facilitate targeted interventions, which may include surgical procedures [18] to correct deformities or address growth plate damage, as well as antibiotic therapy tailored to the specific pathogen involved [19]. Overall, a thorough and multidisciplinary diagnostic approach is fundamental to ensuring optimal outcomes in cases of growth disturbances in osteomyelitis.

Discussion

The management of growth disturbances in osteomyelitis presents a unique set of challenges, particularly in pediatric patients. The complexity lies in the need for a multifaceted approach that addresses both the underlying infection and the skeletal consequences. Surgical interventions, including debridement, epiphysiodesis, guided growth, and corrective osteotomies, have proven effective in correcting deformities and limb length discrepancies [19]. However, the selection of the most appropriate surgical approach should be tailored to the individual patient's needs and specific growth plate involvement. The collaboration of a multidisciplinary team, comprising orthopedic surgeons, infectious disease specialists, pediatric endocrinologists, and physical therapists, is essential to provide comprehensive care [20]. Long-term follow-up and monitoring play a crucial role in assessing treatment efficacy, identifying recurrent infections, and managing potential complications. Patient and family education should remain a focal point in ensuring adherence to treatment regimens and understanding the potential lifelong impact of growth disturbances in osteomyelitis.

## Conclusions

In conclusion, the management of growth disturbances in osteomyelitis demands a multifaceted approach involving timely diagnosis, antibiotic therapy, surgical interventions, physical therapy, and long-term follow-up. Recognizing the importance of addressing both the infection and its skeletal consequences is essential for optimizing patient outcomes and ensuring a better quality of life, particularly in pediatric cases. It is important for clinicians to be aware of growth plate involvement in children with osteomyelitis to initiate prompt treatment and resolution to minimize the long-term effects of growth plate damage.
