# Correction: Disturbance of Growth in Pediatric Patients Due to Osteomyelitis Caused by Growth Plate Infection

**DOI:** 10.7759/cureus.c151

**Published:** 2024-01-16

**Authors:** Jamal Malik, Syed Khaled

**Affiliations:** 1 Department of Research, Liberty University College of Osteopathic Medicine, Lynchburg, USA; 2 Gastroenterology, North Kansas City Hospital, Kansas City, USA

This article has been corrected at the request of the authors to remove the second and third authors who were unaware of the submission and publication of the article and did not provide sufficient contributions to warrant authorship.

